# Mature MiRNAs Form Secondary Structure, which Suggests Their Function beyond RISC

**DOI:** 10.1371/journal.pone.0113848

**Published:** 2014-11-25

**Authors:** Agnieszka Belter, Dorota Gudanis, Katarzyna Rolle, Monika Piwecka, Zofia Gdaniec, Mirosława Z. Naskręt-Barciszewska, Jan Barciszewski

**Affiliations:** Institute of Bioorganic Chemistry, Polish Academy of Sciences, ul. Noskowskiego 12/14, 61-704, Poznan, Poland; H.Lee Moffitt Cancer Center & Research Institute, United States of America

## Abstract

The generally accepted model of the miRNA-guided RNA down-regulation suggests that mature miRNA targets mRNA in a nucleotide sequence-specific manner. However, we have shown that the nucleotide sequence of miRNA is not the only determinant of miRNA specificity. Using specific nucleases, T1, V1 and S1 as well as NMR, UV/Vis and CD spectroscopies, we found that miR-21, miR-93 and miR-296 can adopt hairpin and/or homoduplex structures. The secondary structure of those miRNAs in solution is a function of RNA concentration and ionic conditions. Additionally, we have shown that a formation of miRNA hairpin is facilitated by cellular environment.Looking for functional consequences of this observation, we have perceived that structure of these miRNAs resemble RNA aptamers, short oligonucleotides forming a stable 3D structures with a high affinity and specificity for their targets. We compared structures of anti-tenascin C (anti-Tn-C) aptamers, which inhibit brain tumor glioblastoma multiforme (GBM, WHO IV) and selected miRNA. A strong overexpression of miR-21, miR-93 as well Tn-C in GBM may imply some connections between them. The structural similarity of these miRNA hairpins and anti-Tn-C aptamers indicates that miRNAs may function also beyond RISC and are even more sophisticated regulators, that it was previously expected. We think that the knowledge of the miRNA structure may give a new insight into miRNA-dependent gene regulation mechanism and be a step forward in the understanding their function and involvement in cancerogenesis. This may improve design process of anti-miRNA therapeutics.

## Introduction

Over the last few years, progress in high throughput nucleic acids sequencing resulted in over 30 000 mature miRNAs (over 2 500 human ones) deposited in the miRBase [1]. miRNAs - short (∼20 nt), non-protein coding RNAs [2], [3], regulate the expression of up to 90% of human genes responsible for cell growth, tissue differentiation, cell proliferation, embryonic development, apoptosis and cellular signaling [4]–[7]. They show precise tissue-specific patterns, which may change under pathological conditions [8]–[10], during disease progress and in response to treatment. All of that make miRNAs a bright diagnosis and prognosis factor [11], [12].

MiRNA genes are transcribed to primary miRNA precursors (pri-miRNAs) consisting of up to thousands of nucleotides [13]. They are processed into pre-miRNAs (∼60–70 nt) with ribonuclease Drosha-DGCR8 complex [14]–[16], and exported from nucleus to cytoplasm by Exportin-5. Furthermore, they are cleaved with ribonuclease Dicer into ∼20 nt miRNA duplexes [17], [18]. One of the two RNA strands becomes functional mature miRNA, but the other is released and destroyed [19]. Finally miRNA is incorporated into the miRNA-induced silencing complex (RISC) and targets mRNA in a specific manner [2], [3]. miRNA nucleotides 2-7 (‘seed’ region) are thought to be essential for base pairing with mRNA, its degradation or repression of its translation [20]–[22].

It turned out, that miRNA targets predicted based on the ‘seed’ sequence-match method, such as TargetScan [23], and those gained with the use of proteomic studies are different. Only 50% of the assigned miRNA-target duplexes were confirmed with experimental methods [24]-[26]. Algorithms, based on the energy of miRNA–target binding (e.g. RNAhybrid), structural motifs, such as kissing complexes and the accessibility of target (e.g. mirWIP, PITA) into consideration, gave 80% mutual mRNAs [27].

It has been shown that some mRNA structural motifs, like pseudo-knots, make target site inaccessible for interactions with miRNA, since the energy required to break the existing bonds might be insufficiently compensated by the formation of new bonds with an external molecule, e.g. miRNA [28], [29]. Consequently, miRNA bound to unstructured regions of mRNA, is thought to be energetically favored and that only these targets can be efficiently regulated by miRNAs [29]. The efficiency of miRNA to binding target depends on the miRNA structure within the RISC complex. Argonaute proteins pre-organize miRNA structure, expose ‘seed’ region, and thus facilitate specific recognition of the target mRNA [30]–[32].

However, it is not clear how such a short 8-nucleotide sequence of ‘seed’ region can determine the specificity of miRNA action on a genomic scale. As it has been shown already that seed pairing is not the most reliable predictor [24], we provided data that the miRNA secondary structure affects the miRNA function.

## Methods

### RNA synthesis

The miR-21 (5'UAGCUUAUCAGACUGAUGUUGA), miR-93 (5'CAAAGUGCUGUUCGUGCAGGUAG) and miR-296 (5'AGGGCCCCCCCUCAAUCCUGU) were bought from IBA GmbH, Germany. Pre-miR-21 (5'UGUCGGGUAGCUUAUCAGACUGAUGUUGACUGUUGAAUCUCAUGGCAACACCAGUCGAUGGGCUGUCUGACA) were from Future Synthesis, Poland. Oligonucleotides O1 (5'GTCTGAT), O2 (5'CATCAG), O3 (5'AGTCA), O4 (5'TTCAAC), 05 (5'GAGATTC), O6 (5'ATGAGA), O7 (5'TGGTG), O8 (5'ATCGAC), O9 (5'GATAAGC), O10 (5'CAGTCTG), O11 (5'ATCATGTC), O12 (5'CAACATC) were bought from GenoMed, Poland. The synthesized RNAs and DNAs were purified with standard procedures of PAGE or double HPLC purification. The quality of DNA and RNA was verified in 20% polyacrylamide gels containing 7 M urea.

### [^32^P] labeling of RNA

RNA was labeled at 5'-end with T4 polynucleotide kinase (T4 PNK) (USB) in 10 µl total volume containing 1 µg RNA, 5 µCi [γ-^32^P]ATP, 1xT4 PNK buffer, 0.5 u T4 PNK, at 37°C, 45 min. 3'-end-labeled RNA was prepared with T4 RNA ligase (Fermentas) in 20 µl total volume containing 1 µg RNA, 0.5 mM ATP, 1xT4 RNA ligase buffer, 0.5 u T4 RNA ligase and 6 µl of [5′-^32^P] cytidine 3',5'-bis(phosphate), at 4°C, 16 h [33]. [5′-^32^P] cytidine 3′,5′-bis(phosphate) was synthesized using T4 PNK in 6 µl total volume containing 17 µM cytidine 3'-phosphate, 5 µCi [γ-^32^P]ATP, 1xT4 PNK buffer and 0.3 u T4 PNK, at 37°C, 45 min. Labeled RNAs were PAGE purified.

### Enzymatic probing of RNA

Pre-miR-21, miR-21, miR-93 and miR-296 were partially enzymatically hydrolyzed with RNase T1, RNase V1 and S1 nuclease. Reactions were performed in 10 µl total volume containing 30 000 cpm [5′-^32^P] or [3′-^32^P]RNA, 10 nM RNA and (i) 20 mM CH_3_COONa pH 5.0, 7 M urea, 1 mM EDTA, 0.025 u/µl RNase T1, at 55°C, 20 min. (for limited RNA hydrolysis with RNase T1 in denatured (D) conditions); (ii) 100 mM Tris-HCl pH 7.5, 10 mM EDTA, 0.025 and 0.05 u/µl RNase T1, at 25°C, 15 min (for pre-miR-21 RNA cleavage in native (N1) conditions); (iii) 20 mM CH_3_COONa pH 6.2, 1 mM Mg^2+^, 0.025 and 0.05 u/µl RNase T1, at 25°C, 15 min (for pre-miR-21 RNA cleavage in native-magnesium conditions (N2)); (iv) 100 mM Tris-HCl pH 7.5, 10 mM EDTA, 0.01, 0.02 and 0.04 u/µl RNase T1, at 25°C, 15 min (for miR-21 cleavage in native conditions (N)); (v) 50 mM TrisHCl pH 7.5, 100 mM NaCl, 10 mM MgCl_2_, 0.0002 u/µl RNAse V1 (for limited cleavage of pre-miR-21) and 0.003125, 0.00625 or 0.0125 u/µl RNase V1 (of miR-21 and miR-93), at 25°C, 15 min; (vi) 50 mM CH_3_COONa pH 4.5, 28 mM NaCl, 4.5 mM ZnSO_4_, 0.0095 u/µl nuclease S1 (for limited hydrolysis of pre-miR-21) and 0.00475 u/µl nuclease S1 (of miR-93), at 37°C, 30 min. Reactions were stopped by addition of quenching solution containing 7 M urea, 20 mM EDTA and dyes (0.1% bromophenol blue and 0.1% xylene cyanol). The samples were analyzed with 20% polyacrylamide gels containing 7 M urea electrophoresis. Digestion patterns were visualized by autoradiography. V1 RNase, S1 nuclease and T1 RNase cleavage products in native conditions, were analyzed by comparing them with the OH ladder (50 mM NaOH, 10 mM EDTA, 95°C, 2 min) and ladder obtained from T1 RNase-induced cleavage in denaturing conditions.

### RNase H1 assay

Oligonucleotides (5, 6 and 7-mers), complementary to different regions of pre-miR-21 and miR-21 were used. Reactions were performed in 10 µl total volume containing 20 mM Tris-HCl, pH 7.8, 40 mM KCl, 8 mM MgCl_2_, 1 mM DTT, 1 µM pre-miR-21 or miR-21, 30 000 cpm [^32^P]-labeled pre-miR-21 or miR-21, 5 µM or 10 µM antisense oligonucleotides in reactions with pre-miR21 and 1.25 µM, 2.5 µM, 5 µM and 10 µM with miR-21 and 0.4 u *E. coli* RNase H1 (Fermentas). Samples were incubated at 37°C for 10 minutes, then supplemented with 70 mM EDTA and incubated for 10 minutes, on ice. The reactions were stopped and analyzed as described above.

### Pb^2+^-induced hydrolysis of RNA

Reactions were performed in 10 µl total volume containing 50 mM Tris-HCl, pH 7.5, 10 nM pre-miR-21 or miR-21, 30 000 cpm [^32^P]-labeled pre-miR21 or miR-21 at different Pb^2+^ concentrations (0, 0.1, 0.3, 0.5, 0.7, 0.9, 1.1 mM) for 15 min, at 25°C. They were stopped and analyzed as described above.

### UV melting experiments

miR-21 was melted in a buffer, containing 150 mM NaCl, 10 mM Na_2_HPO_4_/NaH_2_PO_4_, 0.1 mM Na_2_EDTA, pH 6.6. MiR-21 single strand concentrations were calculated from the absorbance above 80°C and single strand extinction coefficients were approximated with the use of the nearest-neighbor model. Absorbance vs. temperature melting curves were measured at 260 nm with a heating rate of 1°C/min from 5°C to 85°C on a JASCO V-650 spectrophotometer with a thermoprogrammer. Melting curves were analyzed and thermodynamic parameters were calculated using the MeltWin 3.5 program.

### Circular dichroism (CD) spectra

CD spectrum of miR-21 was measured in triplicate from 205 to 350 nm at 25°C on a JASCO 815 spectropolarimeter. The buffer was 150 mM NaCl, 10 mM Na_2_HPO_4_/NaH_2_PO_4_, 0.1 mM Na_2_EDTA, pH 6.6 and the miR-21 concentration was 13.2 µM. The measured CD spectrum was averaged, spectrum of the buffer subtracted and the result converted into molar ellipticity per nucleotide (Δ*ε*). MiR-21 was melted in the same buffer. M_deg_ vs. temperature melting curves were measured at 260 nm with a heating rate of 0.5°C/min from 10 to 75°C on a JASCO V-650 spectrophotometer with a thermoprogrammer. Melting curves were analyzed and 1st derivative was calculated with the Origin 6.0 program.

### NMR spectra

For studies of the exchangeable protons, the solvent was H_2_O/D_2_O (9∶1, v/v). The samples were annealed by heating at 90°C for 5 min and then slowly cooled down to the room temperature and stored at 4°C. 1D proton NMR spectra were recorded using different concentrations of miRNAs and salt as well as various temperatures. For 2D NMR experiments all miRNAs were dissolved in 150 mM NaCl, 10 mM Na_2_HPO_4_/NaH_2_PO_4_, 0,1 mM EDTA, pH 6.6. The final RNA concentration of miR-21 was 0.70 mM and of miR-93 was 0.75 mM.

NMR spectra were collected on a Bruker AVANCE III 700 MHz spectrometer, equipped with a QCI CryoProbe. The 3 mm thin wall tubes were used with a final sample volume of 220 µl. The water signal was suppressed by excitation sculpting with gradient pulse. The assignment of resonances was based on homonuclear ^1^H-^1^H NOESY and heteronuclear ^1^H–^15^N HSQC experiments. The ^1^H-^15^N HSQC spectrum was collected from 1216 scans at 7°C and ^1^H-^1^H NOESY spectrum was collected from 120 scans with 150 ms mixing time at 15°C for miR-21 sample. The ^1^H-^15^N HSQC spectrum was collected from 992 scans at 15°C and ^1^H-^1^H NOESY spectrum was collected from 88 scans with 150 ms mixing time at 15°C for miR-93 sample. Spectra were processed and prepared with TopSpin 3.0 Bruker Software.

### Concentration calculation of individual miRNA

The average concentration of individual miRNA was calculated based on published data. The median copy number of individual miRNA in a single cell is ∼200 [34] and an average volume of mammalian cell (HeLa) is 2×10^−12^ dm^3^ [35]. 6.022×10^23^ is 1 mole, the 200 miRNA copies present 33.21156×10^−23^ mole. If 33.21156×10^−23^ mole is in 2×10^−12^ dm^3^, therefore in 1 dm^3^ is 166 pmol, thus the average concentration of an individual miRNA is 166 pM.

### Calculation of miRNA hairpin and homoduplex equilibrium

miRNA monomer and homoduplex concentration dependency plots was determined using RNAcofold (http://rna.tbi.univie.ac.at/cgi-bin/RNAcofold.cgi) [36]. The output shows of the minimum free energy (mfe) structures of miRNA in dimer and monomer in bracket notation, base pairing probability matrix (using the -p switch) and plot illustrating functions of monomer and dimer partition of RNA concentration. From functional equations, fractions of miR-21, miR-93, miR-296 particles in dimer and monomer, in particular RNA concentration, were calculated.

### miRNA stability in GBM lysate

[^32^P]-radiolabeled miR-21, miR-93 and miR-296 were incubated in glioblastoma multiforme tissue lysate (protein concentration: 0.01 mg/ml), at 37°C, for 15, 30, 60, 120 and 180 min. In the case of miR-296, the incubation was prolonged to 8, 15, 24 and 40 h. The reactions were stopped and analyzed as described above. The half-life of miRNAs in GBM lysate was calculated with the use of GraphPad Prism.

### Library construction

All human miRNAs sequences available on 17^th^ October 2012 were downloaded from miRBASE version 19. The data were scanned for unique sequences of mature miRNAs. The molecules with identical sequences, but annotated under different names in the miRBASE were removed. Finally, the curated library contained 2042 sequences of human miRNA.

### *In silico* analysis

Mfold program version 3.5 (http://mfold.bioinfo.rpi.edu) was used to calculate structures of all human miRNAs and to evaluate the folding free energy of miRNA hairpins at 37°C and 1 M NaCl. ModeRNA (http://iimcb.genesilico.pl/moderna/) was used for 3D miRNAs structure modeling based on the experimentally confirmed structures of short RNAs as templates. Additionally, RNAmetaserver (http://iimcb.genesilico.pl/rnametaserver), which provides access to single-sequence (20 programs) and comparative methods (10 programs) predicting RNA secondary structure, was used to miR-21, miR-93, miR-296 secondary structure predictions. The RNAfold web server was used to predict the minimum energy structure and of a complete set of suboptimal structures of single stranded RNA [37]. The similarity of miRNA structures obtained with biochemical and spectroscopic methods and anti-Tn-C aptamer TN-9.6 was calculated using RNAforester [38].

### Data analysis

All experiments were repeated at least three times unless otherwise stated. The products of chemical and enzymatic structure probing of miRNAs were visualized with a Fuji Film FLA 5100 phosphoimager using the manufacturer's (Fuji) software and quantified using the ImageQuant software (Molecular Dynamics). Cleavage yield was estimated by treating the density of the control band as 100% and calculating the density of the product band as x %. The half-lives of miRNAs in GBM lysate were calculated with GraphPrism (nonlinear regression, one phase exponential decoy equation).

## Results and Discussion

### Majority of miRNAs have intrinsic potential to form secondary structures

Being interested in brain tumors, some time ago we have found tenascin-C (Tn-C) as a new marker and target of GBM therapy, and developed double-stranded RNA (ATN-RNA) intervention for its silencing [39]. Recently, we have shown that the level of some miRNAs is changed in GBM in comparison with normal tissue. For example, miR-21, miR-93 and miR-296 are significantly overexpressed in gliomas. Both Tn-C and these miRNAs modulate glioblastoma cells adhesion, migration and growth and their level correlate well with the tumor grade. To unravel the mystery of this deadly brain tumor, we decided to determine miR-21, miR-93 and miR-296 structure and asked about a relation between Tn-C and these miRNAs. As miRNAs are markers of numerous diseases and prosperous targets of their therapy, the structure of mature miRNA may give a new insight into orchestred miRNA-dependent gene regulation and be a step forward to understand miRNAs functions, their involvement in cancerogenesis and improve designing anti-miRNA therapeutics.

**Figure 1:**
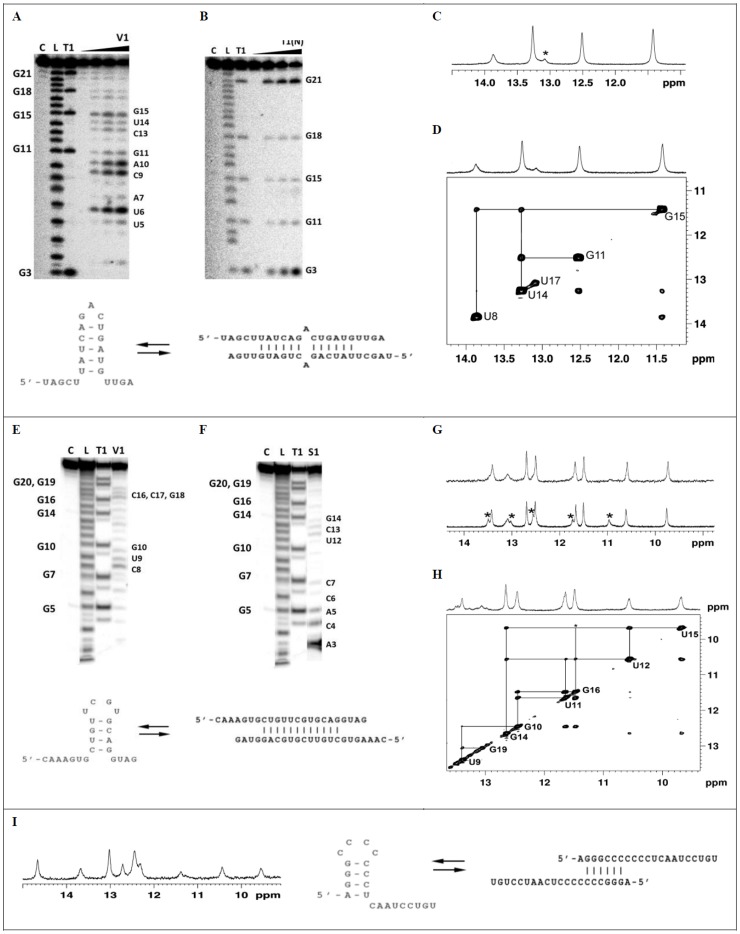
Enzymatic probing (A, B, E, F) and NMR analysis (C, D, G, H, I) of miR-21 (A-D), miR-93 (E-H) and miR-296 (I). **A, B, E, F**. Cleavage patterns obtained for limited hydrolysis of 5'-end labeled miR-21 (**A, B**) and miR-93 (**E, F**) with RNase V1, nuclease S1 and RNase T1 in native conditions. Lanes: C - reaction control; L – OH ladder; T1 - limited hydrolysis by RNase T1 (0.025u/µl) in denaturing condition. **A**. Lines V1 - limited hydrolysis with RNase V1 (0, 0.03125, 0.0625 or 0.125 u/µl). **B**. Lanes T1(N) - limited hydrolysis with RNase T1 (0, 0.04, 0.02 or 0.01 u/µl) in native conditions. **E**. Line V1 - limited hydrolysis with RNase V1 (0.125 u/µl). **F**. Lines S1 - limited hydrolysis with nuclease S1 (0.00475 u/µl). The increasing concentrations of RNase V1 and RNase T1 are indicated by arrows. Cleavage sites are indicated in autoradiogram. **C, D, G, H, I**. NMR analysis of miR-21 (**C, D**) miR-93 (**G, H**) and miR-296 (**I**) structures. **C**. The imino region of ^1^H NMR spectrum of miR-21 (0.7 mM) recorded at 7°C in H_2_O:D_2_O (90%:10%) with 150 mM sodium chloride, 10 mM phosphate buffer and 0.1 mM EDTA. Resonances arising from the hairpin form are indicated with *. **D**. Imino region of the ^1^H-^1^H 2D NOESY spectrum of miR-21 at 15°C in H_2_O:D_2_O (90%:10%) with 150 mM sodium chloride, 10 mM phosphate buffer and 0.1 mM EDTA. The lines indicate the imino proton connectivity. **G**. The imino region of ^1^H NMR spectrum of miR-93 (0.75 mM) recorded at 25°C in H_2_O:D_2_O (90%:10%) with 150 mM sodium chloride (top) or 50 mM sodium chloride (bottom), 10 mM phosphate buffer and 0.1 mM EDTA. Resonances arising from the hairpin form are indicated with *. **H**. Imino region of the ^1^H-^1^H 2D NOESY spectrum of miR-93 at 15°C in H_2_O:D_2_O (90%:10%) with 150 mM sodium chloride, 10 mM phosphate buffer and 0.1 mM EDTA. The lines indicate the imino proton connectivity. **I**. The imino region of ^1^H NMR spectrum of miR-296 (0.3 mM) recorded at 25°C in H_2_O:D_2_O (90%:10%) with 150 mM sodium chloride, 10 mM phosphate buffer and 0.1 mM EDTA.

Using specific probes, such as RNase T1, RNase V1 and S1 and spectroscopic methods, such as NMR, UV/Vis and CD spectroscopies, we analyzed miR-21, miR-93 and miR-296 structures (**Figs. 1** **, S1** and **S2**). In order, to get a deep understanding of specificity and susceptibility of these methods applied to studies of RNA structure and its slight fluctuations, we also mapped the structure of miR-21 precursor (pre-miR-21) (**Figs. S3–S5**).

#### miR-21

miR-21 was efficiently cleaved with RNase V1 within U6U7, C9A10G11 and C13G15 regions, with S1 nuclease especially within G18U19U20G21 residues (**Fig. 1A**). RNA was hydrolyzed with T1 RNase at all guanines (G3, G11, G15, G18, G21) in denatured conditions. However in native conditions, G11, G15 and G18 are slightly less cleaved with T1 RNase than other guanines residues (**Fig. 1B**). DNA oligonucleotides complementary with miR-21 induce its cleavage with RNase H1. The differences in RNase H1 activity showed that miR-21 regions complement to O1 and O11 are less prompt to complex oligodeoxynucleotides (**Figs. S1A, S1B**). The RNase T1 and H1 hydrolysis patterns of miR-21 obtained with indicate that it forms secondary structure. It can be hairpin with five base pairs stem, three nucleotide loop and 3' and 5' dandling ends or imperfect homoduplex with free ends and mismatch in the central part (**Fig. 1, A-D**). Because of the involvement of the same base-pairs in hydrogen bonding, in the both forms, this approach does not allow to distinguish between miR-21 hairpin and homoduplex. Therefore, we established miR-21 secondary structure using NMR spectroscopy (**Figs. 1C, 1D**). The presence of five imino resonances in the region of 11.5–14.3 ppm of the ^1^H NMR spectrum reflects the formation of five Watson-Crick base pairs (**Fig. 1C**). The chemical shifts of the imino protons were almost independent of the concentration of miR-21 (0.27 µM and 0.7 mM) and salt (50 mM and 150 mM) (**Fig. S1E**). Any additional signals which would suggest the formation of another, less stable structural form were not observed at low temperatures (**Fig. S1F**). ^15^N chemical shifts of the attached nitrogen showed that the resonance between 13–14 ppm correspond to the uridine imino protons and that between 11.4–12.8 ppm to the guanosine imino protons (**Fig. S1C**). The NOESY spectrum recorded in H_2_O∶D_2_O (9∶1, v/v) showed the typical pattern characteristic of Watson-Crick base pairs (**Fig S1D**). The melting temperature determined for miR-21 by UV (**Fig. S1G**) and CD (Figs. S1H, S1I, S1J) depends on RNA concentration, which indicates that under experimental conditions miR-21 forms duplex. However, the occurrence of miR-21 hairpin at low miRNA concentration should not be excluded.

#### miR-93

C8U9G10 and C16C17G18 residues of miR-93 were efficiently cleaved with RNase V1 (**Fig. 1E**), but A3C4A5C6C7 and U12C13G14 with nuclease S1 (**Fig. 1F**). The hydrolysis patterns indicated that miR-93 forms either hairpin with four nucleotide double stem and four nucleotide loop, or duplex with 3' and 5' dandling ends. However, the observed hydrolysis at U12, C13 and G14 residues with both RNase V1 and nuclease S1 suggests that both structural forms of miR-93 coexist (**Fig. 1E,F**). Finally, miR-93 secondary structure was confirmed with NMR spectroscopy. Two sets of resonances in the imino region of ^1^H NMR spectrum were visible in the presence of 50 mM NaCl at 25°C (**Fig. 1G**). At 150 mM NaCl, the imino signals of the minor form disappeared and only resonances corresponding to the major form were detected. ^1^H-^15^N HSQC spectrum was used to distinguish the G and U imino protons of the major form of miR-93 (**Fig. S2A**). The analysis of the NOE contacts between the imino-imino (**Fig. 1H**) and imino-amino protons (**Fig. S2B**) allowed to define the region of the structure between C8 and G19 as double stranded. The strong NOE correlation observed between resonances at 10.55 and 9.7 ppm attributed to U12 and U15 uridine imino protons, respectively, suggested the formation of U12:U15 mismatch. Another strong NOE cross-peak, observed between G16 and U11 imino protons, was assigned to G:U mismatch. The formation of the stable G14:C13 Watson-Crick base pair was confirmed by the analysis of the sequential and cross-strands NOE connectivities of imino signal at 12.65 ppm assigned to G14 residue (**Fig. 1H**). The presence of U12:U15 mismatch and a stable G14:C13 Watson-Crick base pair (**Fig. S2C**) unambiguously identified miR-93 duplex as the major form under experimental conditions. In a hairpin structure residues from U12 to U15 constitute the loop for which different pattern of NOEs would be expected. We could not unambiguously assign the resonances of the minor form observed at 50 mM NaCl, however, they most probably correspond to the hairpin form.

#### miR-296

Enzymatic hydrolysis of miR-296 with RNase V1, T1 and nuclease S1 indicated that this molecule acquire secondary structure. Enzymatic probing does not allow to discriminate between miR-296 hairpin and duplex. ^1^H NMR spectrum shows that two forms of miR-296, hairpin and homoduplex, coexist in solution (**Fig. 1I**). The hairpin form with four base pair stem, four nucleotides loop and nine nucleotide unpaired region at the 3'-end, as well as homoduplex with six base pairs double stranded region at 5'-end and nine nucleotide free region at the 3'-end of the molecule are possible (**Fig. 1I**). In summary, the biochemical, UV, CD and NMR data showed that the analyzed mature miR-21, miR-93 and miR-296 have an intrinsic potential to form secondary structures, both hairpin and homoduplex and that hairpin-duplex equilibrium is concentration dependent.

#### Pre-miR-21

Pre-miR-21 structure was also mapped to get a deep insight into the susceptibility of biochemical methods to RNA hairpin structure (**Figs. S4–S5**).

C4-C16, U21-G32, C52-U55 and U64-U68 residues of pre-miR-21 were cleaved with RNase V1. The regions A42-C51 and C56–C63 and A17, A23 and A29 residues, which were thought to be in double stranded region, were not susceptible to RNase V1. G35-U60 residues were efficiently cleaved with nuclease S1. Pre-miR-21 was cleaved at all guanine residues by RNase T1 in denatured condition. T1 RNase in both native and native-magnesium conditions strongly hydrolyze at G35 residue, but guanines G32, G44 and G45 with less efficiency (**Fig. S3**).

For H1 ribonuclease cleavage, out of eight designed oligonucleotides (O1 – O8) complement of different regions of pre-miR-21, only one (O5), complementary to G35-C41 region bound to pre-miR-21 and formed the duplex cleaved with RNase H1 (**Fig. S4**). The hydrolysis patterns of pre-miR-21 after Pb^2+^ treatment revealed one prominent site-specific cleavage in the pre-miR-21 (G32-A42) and several minor cleavage sites. G32-A42 residues comprised the strong Pb^2+^ binding site with especially efficient cleavage within the A36-U40 region. At higher Pb^2+^ concentrations (0.7–1.1 mM), the terminus of pre-miR-21 and the residues in a close proximity of A17 bulge of the hairpin stem, also within its complementary strand - region G54-U59, were cleaved (**Fig. S5**).

The results proved that pre-miR-21 forms hairpin and that RNase V1, T1 and nuclease S1 are sensitive and reliable for RNA structure analysis. As obtained results confirmed specificity and susceptibility of used methods, we did not mapped other pre-miRNAs.

#### *In silico* analysis of human miRNA structure

**Figure 2:**
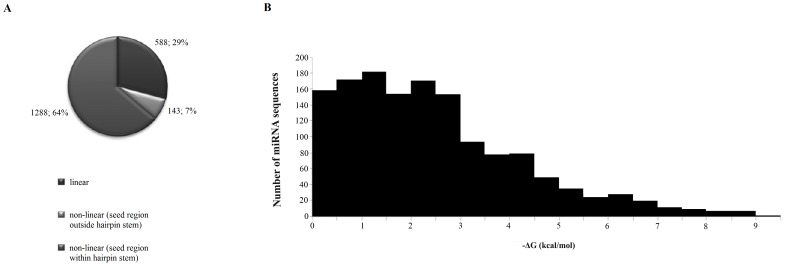
Distribution of linear and nonlinear human miRNAs and of hairpin stem location in predicted human secondary structure (A); and folding distribution of human mature miRNAs strands (B).

To determine the propensity of other human miRNAs to form secondary structures we used bioinformatics tools. First of all we established the cured library of 2042 sequences of human miRNAs, which was used for further analysis. Our *in silico* studies showed that over 75% of human miRNAs may form both hairpin and homoduplex (**Fig. 2A**). The most widely represented miRNA loops are, as follows: tetraloop, threeloop, fiveloop, found in 433, 413 and 283 non-linear miRNA structures, respectively. Within numerous miRNA sequences, we found UUCG, GAAA, GCAA, GAGA, GUGA, GGAA, CUUG, UUUG motifs, known as nucleation sites for hairpin folding [40]. They determine compact shape and stability of hairpins [40]. The most widely represented motif in human miRNA sequences is GGAA tetranucleotide, although motifs GAGA, GAAA and GUGA motives are also abundant. We showed that in many cases they border with at least 3 nucleotide stems and GC closing pairs. Thus, we conclude that these miRNAs display a high probability to form stable hairpin structure. The minimal free energy span of predicted miRNA hairpins is very wide, from −0.1 to −11.1 kcal/mol. The most structures show −0.1 to −3 kcal/mol (**Fig. 2B**).

Our results are consistent with previous *in silico* studies, which have also shown that above 70% of human [41], mammalian [42] and plants [43] miRNAs may fold into hairpin structures and almost 70% could potentially form self-aggregated homoduplexes.

### miRNA concentration within the cell

The average concentration of individual miRNA was calculated, taking into account the fact that the median copy number of individual miRNA in a single cell is ∼200 [44] and that an average volume of mammalian cell (HeLa) is 2×10^−12^ dm^3^ [35]. Thus, the average concentration of an individual miRNA is 166 pM. The obtained figure is significantly different from the published data, which have shown that an average miRNA concentrations in a typical animal cell can be 2.2 mM (1000 miRNA copies in a 1000 mm^3^ cell), or even exceed 22 mM (10,000 copies in a 1000 mm^3^ cell) [34].

### miR-21, miR-93 and miR-296 structure depends on miRNA concentration

As nucleotides are the polar and negatively-charged molecule, oligonucleotides to shield they electronegative potential of phosphate groups, fold and/or associate with positively charged molecules, mainly cation ions. As the majority of miRNAs have self-complementary regions, they exist as self-complementary hairpin and/or homo-duplex structures in solution. The partitioning of individual nucleic acid molecules among intra- and intermolecular conformations is governed by both kinetic and thermodynamic factors, and in general depends on ionic conditions and miRNA concentration [41].

**Figure 3:**
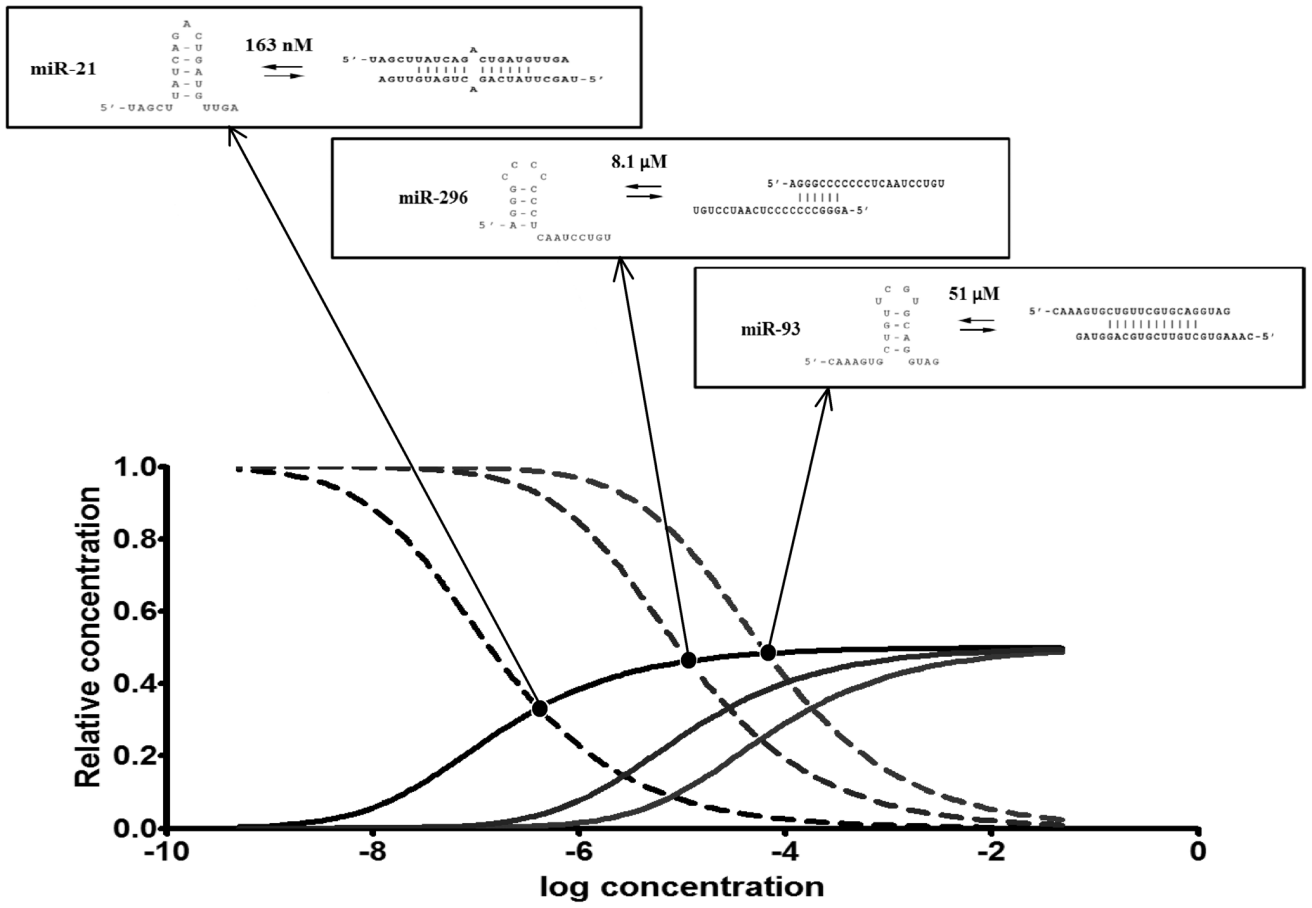
Concentration dependency plots of miR-21, miR-93, miR-296 monomer and dimer (A) and the scheme illustrating concentration dependent equilibriums of these miRNAs (B).

We used miRNAcofold to calculate hairpin-homoduplex equilibrium for mR-21, miR-93 and miR-296 and to compute the partition of miRNA hairpin and duplex in cellular concentration of miRNA. The results are showed in the **Fig. 3** and **Table 1**. The equilibrium of hairpin and homoduplex of miR-21, miR-93, miR-296 depends on the miRNA concentration (**Fig. 3**). It is consistent with previous data, concerning other RNAs [41],[44]. At high concentration of RNA, duplexes are formed, but in lower ones, hairpins constitute the prevailing form [41].

**Table 1:** Some parameters of miRNAs and the partition of monomer and dimer formation in concentration dependence.

	miR-21	miR-93	miR-296
Free energy of dimer [kcal/mol]	−13.458649	−13.541779	−15.170716
Free energy of monomer [kcal/mol]	−1.980884	−3.900905	−4.140155
Concentration in which miRNA dimer and monomer are in equilibrium [M]	163 nM	51 µM	8.1 µM
Concentration in which 50% of molecules form dimer [M]	135 nM	60.7 µM	9.27 µM
The partition of monomer fraction in 10 nM miRNA	88.5%	∼100%	∼100%
The partition of monomer fraction in 0.7 mM miRNA	1.9%	31.5%	14.5%
The partition of monomer fraction in cellular miRNA concentration (166 pM)	∼100%	∼100%	∼100%

From functional equations, fractions of miR-21, miR-93, miR-296 occur in dimer and monomer, in particular RNA concentrations, were calculated. miRNA concentration, in which miRNA hairpin is in equilibrium with homoduplex are 163 nM, 51 µM and 8.1 µM for miR-21, miR-93 and miR-296 respectively. It explains why, under experimental conditions, (NMR, CD, UV, enzymatic probing) both miRNA hairpins and homodimer were observed. In NMR, CD and UV spectroscopy (∼0.7 mM miRNA) we observed miRNA dimer as the prevailing form of miRNA. In 0.7 mM concentration of miRNA, 98.1, 68.5 and 85.5% miR-21, miR-93 and miR-296 occur as dimers. In lower miRNA concentrations, under enzymatic probing experiments (10 nM miRNA) also both forms were observed, but the equilibrium was shifted to miRNA monomers (hairpin). From functional equations, in 10 nM RNA concentration, miRNA hairpins are the prevailing form and establish 88.5% for miR-21 and almost 100% both for miR-93 and miR-296 (**Fig. 2**, **Table 1** **)**. We would underline that in 166 pM miRNA (average cellular miRNA concentration), monomer accounts for almost all of these miRNA.

The folding of all human mature miRNAs, performed in physiological conditions and our experimental studies showed miRNA hairpins formation is facilitated by a cellular environment. We presume that every fluctuations of cellular miRNA concentration and ionic conditions may result in the hairpin-to-duplex transition. Recently, it has been shown that within the cell, the total miRNA is in excess to Ago 1-4 proteins [45]–[47]. The finding indicates that miRNA may exist in the cell as a free molecule [46], [47]. Thus we expect that within the cell, majority of miRNAs which are not knotted in RISC complex, exist as hairpins.

### miRNA stability depends on its structure

**Figure 4:**
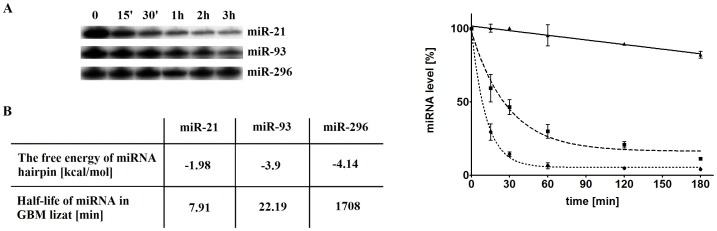
miR-21, miR-93 and miR-296 stability in GBM lysate. **A, B**. Hydrolysis of 5′-end labeled miR-21, miR-93 and miR-296 in 0.01 mg/ml GBM lysate. **C**. Half-lives of miR-21, miR-93 and miR-296.

We checked whether the structure has an impact on miRNA stability. We determined half-lives of miR-21, miR-93 and miR-296, a glioma-specific miRNAs in cell lysates prepared form glioblastoma multiforme tissues. We noticed that the structure indeed influences miRNA stability and their surveillance in cellular-like environment. We observed significantly longer half-life of miR-296 in comparison to others miRNA and that miRNA stability correlates with ΔG of their structure (**Fig. 4**). Additionally, the fact that 5'-end of miR-296 is knotted in the hairpin stem may explain the high stability of this miRNA. The differences of secondary structure energy may explain the distinct turnover time for the given miRNAs. Our results are fully supported by other data, which have shown various stability, resistance to nucleases and surveillance in the cell for different miRNAs [48]. It has been shown that an average half-life of miRNA is about 119 h (i.e. 5 days), ranging from 72 h–225 h, but some miRNAs fluctuate more rapidly than others, e.g. miR-155 appeared to be less stable [41], [48], [49]. It is widely known that the RNA structural motifs contribute to RNA stability [50], so it is not surprising and easy to conduct based on the general knowledge, that miRNA structure determines its stability. However, till now no direct evidences for that have been provided. We, for the first time, correlated miRNAs stabilities with their structures. Previously, some other factors determining miRNA stability have been known, such as Ago2 [51] and GW182 [52], [53]. Especially, Ago-dependent stability is a common feature of mammalian miRNAs [54]. Both Ago2 and GW182 response for assembly of miRNA-induced silencing complex, protect miRNAs from degradation by RNases, and thus response their turnover [52].

### Structural similarity of miR-21, miR-93 and anti-tenascin C aptamer TN-9.6

**Figure 5:**
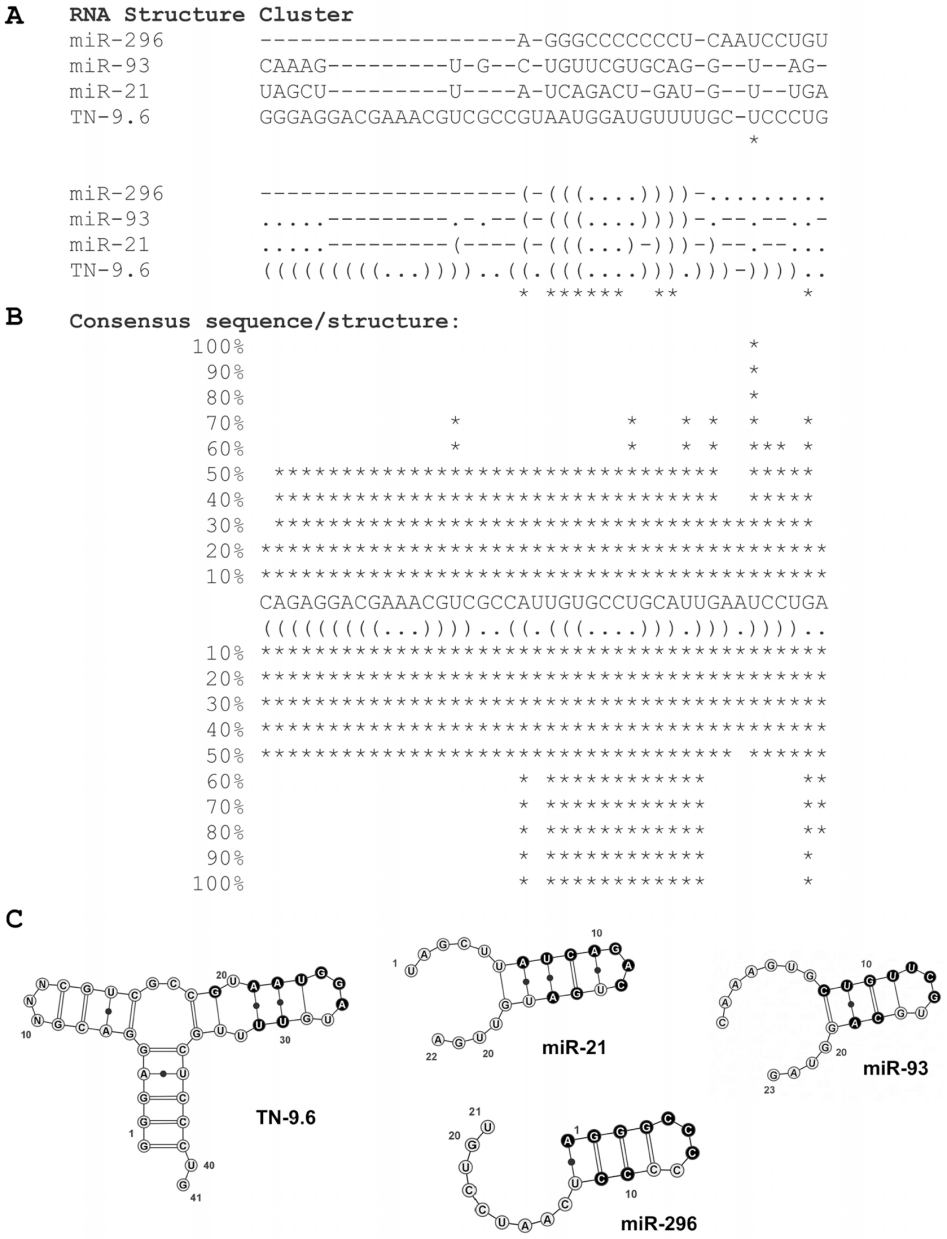
Similarity of structures of miR-21, miR-93, miR-296 and anti-Tn-C aptamer TN-9.6. **A**. RNA structure cluster of miR-21, miR-93, miR-296 and TN-9.6. **B**. ASCII representation of a consensus sequence and structure of miR-21, miR-93, miR-296 and TN-9.6. Multiple structure alignments of anti-Tn-C aptamer TN-9, its derivatives (TN-9.4, TN-9.6), miR-21 and miR-93 based. **C**. Graphic representation of miR-21, miR-93, miR-296 and TN-9.6 structures similarity. Regions of structures identical within compared group are marked in black. Calculations are made by RNAforester algorithms. The obtained score is 80.25.

Looking for functional implications of miRNA and taking into account that miRNA hairpins resemble aptamers, which form stable structure composed of hairpins, we performed multiple sequence and multiple structure-based alignments of miRNAs and anti-tenascin C aptamers. Strong overproduction of miR-21 and Tn-C is observed in GBM, which may suggest direct correlation between them. The multiple structure aligment of miR-21, miR-93, miR-296 and TN-9.6 calculated by RNAforester showed us high similarity of structures of these miRNAs and anti-Tn-C aptamer (**Fig. 5**). It has been shown earlier that TN-9 aptamer truncated at 3′ end and 17 nt (positions 10–27) replacement of TN-9.4 with a single (CH_2_CH_2_O)_6_) does not cause affinity to tenascin C loss, indicating that the short hairpin of TN-9.6 is sufficient for interaction with fibronectin type III repeats 3-5 of Tn-C [55]. The high structure similarity of TN-9.6 hairpin to miR-21, miR-93 and miR-296 hairpins leads to the assumption that miRNAs, and other short RNA hairpins, may directly modulate protein activity

The idea is brand new, so there are only few, however strong evidences supporting our observations that miRNAs may function beyond RISC. For example, miR-888 and miR-146a have been shown to bind to the nucleocapsid domain of the Gag protein, the main structural component of HIV-1 virions and interfere with viral-RNA-mediated Gag assembly and viral budding at the plasma membrane [56]. Interestingly there are known RNA aptamers, which target the same viral peptide [57], [58]. It has been shown that anti-Gag RNA aptamers, which as mentioned above miRNAs, target HIV-1 Gag protein and inhibit virus production [57], [58]. Additionally some algorithms to *in silico* structure modelling predict that these miRNAs may form secondary structure, both hairpins and homodulexes. It is a direct evidence that miRNAs may function exactly in the same way as RNA aptamers.

The other mature miRNAs targeting proteins outside the RISC complex are the members of miR-1/miR-206 family members [59]. They interact with TDP-43, an RNA-binding protein that aggregates in individuals afflicted with amyotrophic lateral sclerosis [59]. Till now the function of these interaction was not determined. It is postulated that TDP-43 inhibit miRNAs functions, however the other way round, TDP-43 inhibition by these miRNAs, may not be excluded.

Recently, slightly different example of miRNA regulatory function beyond RISC has been described [60]. Mature mouse miR-709 predominantly located in the nucleus, recognizes and binds pri-miR-15a/16-1 preventing its processing into pre- miR-15a/16-1, and thus regulation of miR-15a/16-1 maturation.

The above mentioned results support our idea that miRNAs may function as a regulatory molecule also beyond the RISC complex. One can noticed that more and more researchers are looking beyond non-canonical function of miRNAs.

## Conclusions

The current model of miRNA action does not explain how such a short nucleotide sequence of ‘seed’ region can determine the specificity of miRNA action. Thus using specific nucleases (RNase T1, RNase V1 and nuclease S1), NMR, UV/Vis, CD spectroscopies, we showed that miR-21, miR-93 and miR-296 form secondary structure depending on miRNA concentration hairpin and/or homoduplex. We found that at low cellular concentration of miRNA, the equilibrium of these forms is shifted to RNA hairpin, and thus we do postulate that within the cell miRNA hairpins are more abundant than its dimers. Considering miRNA concentration and also its structure in the cell, it should be taken into account that the majority of miRNA is knotted into RISC complex and miRNAs which escaped from the complex or are a product of non-canonical miRNA biogenesis pathway may exist and function as free molecules. The additional argument to support this idea is the excess of total miRNAs, related to Ago1-4 proteins has been shown [46], [47].

miRNA structure affects its turnover and function. We indicated that miRNA structure determines its stability, resistance to nucleases and surveillance in the cell. We noticed that miRNAs, which fold into knotty, secondary structures are more stable and that miRNA stability correlates with ΔG of their structure. Additionally, we observed that the half-life of miRNAs is longer when their ends are knotted in hairpin stem, e.g. like in the case of miR-296. The high-order motifs within miRNA and the differences in secondary structure energy may explain the different turnover time of miRNAs. It suggests that a of novel mechanism, regulating miRNA function through fine-tuning of steady-state miRNAs level, exists.

Previously, it has been reported that the miRNA binding availability to target mRNA is highly dependent on mRNA structure [29]. The structural motif, like pseudo-knots, comprise a target site closed and inaccessible for interactions with miRNA, since the energy required to break the existing bonds might be insufficiently compensated by formation of new bonds with an external molecule, in this case the miRNA [28], [29]. Consequently, it is thought that miRNA binding to unstructured regions of mRNA is energetically favored and that only these targets can be efficiently regulated by miRNAs [29]. Additionally, there is also some evidence that the secondary structure of mature siRNA also influences the efficiency of siRNA-mRNA interaction, where unstructured siRNA confers stronger silencing abilities than structured guide siRNAs. By analogy to siRNA, we thought that that the stable self- and duplex miRNA structures may influence miRNA-mRNA complex formation. Thermodynamic of RNA-RNA interactions depends both on the structure and accessibility of mRNA target sites and structure of regulatory miRNA as well. The secondary structure of miRNA can have a conformational role to modulate miRNA-mRNA interactions and thus can explain the different degree of genetic regulation of the specific miRNA involved in regulation process. It has been shown that bases 2-7 of 5′ end of the miRNA are crucial to initiate mRNA binding. Thus, we suppose that miRNAs, which seed regions are not knotted in secondary structure, interact more easily with target mRNA than those, which seed region is a part of hairpin stem, like e.g. in the case of miR-93 and miR-296.

Interestingly, analysis of chromatin-bound Argonaute proteins identified multiple AGO-associated splicing factors, also miRNAs demonstrating a direct link between miRNAs and chromatin remodeling [61]. The formation of a ternary complex between the target transcript, miRNAs and Ago proteins might conceal splicing recognition motifs, thereby precluding binding of splicing factors and modulating pre-mRNA splicing events [61]. There are also other examples, which support the hypothesis that miRNAs contribute to the regulation of alternative splicing. miR-320, recruits AGO1 and EZH2 to the *POLR3D* promoter, which induces silent state chromatin formation [62]. Similarly, miR-709 and miR-233 recruit Argonaute-1 to respectively *Egr2* and *NF1A* promoters, which results in chromatin remodeling and silence of these genes [63], [64].

Looking for new functional consequences of miRNA structure we perceived that structure of miRNA hairpins resemble aptamers, which are also small oligonucleotides with high affinity and specificity for their target molecules. For the comparison we have chosen anti-tenascin C (anti-Tn-C) aptamers, which inhibit brain tumor glioblastoma multiforme (GBM, WHO IV) through the interaction with Tn-C and selected miRNA hairpins. All studied miRNAs are highly overexpressed in gliomas. This strong overproduction of miR-21, miR-93 and Tn-C observed in GBM implies some connection between them. The structural similarity of these miRNA hairpins and anti-Tn-C aptamers indicates that miRNAs may function also beyond RISC and be even more sophisticated regulators, that it was previously expected.

There are only few, however strong evidences supporting our observations miRNAs may function exactly in the same way as RNA aptamers. We noticed that more and more researchers are looking beyond non-canonical function of miRNAs. Now there are only few evidences supporting our thesis, however we suspect more in nearest feature.

In summary, we showed that miRNAs form both hairpins and homodulexes, presented a high structure similarity of some miRNAs hairpins to aptamers and concluded that structural versatility of miRNA may determine a variety of functions beside widely accepted mRNA recognition, degradation and destabilization. We suggest that miRNAs, as RNA aptamers, may be prompted to interact with proteins, and consequently directly regulate their activity. Thus, we postulate that miRNA hairpins may function beyond the miRISC, both in a sequence and structure dependent manner.

## Supporting Information

Figure S1 **Enzymatic (A, B) probing of miR-21 structure, NMR analysis (C-F), UV (G) thermal melting and circular dichroism profile (H-J) of miR-21.** **A, B**. Cleavage patterns obtained for limited hydrolysis of 5'-end labeled miR-21 with RNase H1 in presence of oligodeoxynucleotides complement to different regions of miR-21, and ‘hammerhead’ ribozyme. Lanes: C - reaction control; L – OH ladder; T1 - limited hydrolysis with RNase T1 (0.025 u/µl) in denaturing condition. **A**. The sequences of oligonucleotides (O1, O9-O12) complement to different regions of miR-21. Sequence of miR-21 is marked in grey. **B**. Lines O1, O9-O12 – hydrolysis with RNase H1(0.04 u/µl) in different concentrations (0, 1.25, 2.5, 5 or 10 µM) of oligonucleotides (O1, O9-O12) complement to different regions of miR-21. **C**. ^1^H-^15^N HSQC spectrum of the miR-21 recorded at 7°C. The assignments are indicated. **D**. Expanded 2D NOESY contour plots (150 ms mixing time) of miR21 (0.7 mM) at 15°C. The cross peaks *a* to *f* are assigned as follows: a – U8:NH1-A16:H2, b – U14:NH1-A10:H2, c – G11:NH1-C13:NH4_2_ d - G11:NH1-C13:NH4_1_, e - G15:NH1-C9:NH4_2_, f - G15:NH1-C9:NH4_1_. **E**. Imino regions of the 1D ^1^H spectra recorded at different RNA strand and salt concentrations. Assignments are annotated for the imino proton resonances. **F**. Imino region of the 1D ^1^H spectra of the miR-21 recorded at various temperatures. Assignments are annotated for the imino proton resonances. **G**. Analysis of T_m_ dependence over the ranges 0.8–73 µM. **H, I**. CD thermal melting profiles of miR-21 (4.2 µM), (**H**) (22.6 µM), (**I**) at 260 nm obtained for the temperatures between 10°C–75°C. The melting temperatures of approximately 35°C and 43°C were estimated from the first derivative. **J**. Circular dichroism (CD) profile of miR-21 (13.2 µM) obtained at 25°C (150 mM sodium chloride, 10 mM phosphate buffer and 0.1 mM EDTA, pH 6.6).
(TIF)

Figure S2 **NMR analysis of miR-93.** **A**. ^1^H-^15^N HSQC spectrum of the miR-93 recorded at 15°C. The assignments are indicated. **B**. Expanded 2D NOESY contour plots (150 ms mixing time) of miR-93 molecule (0.75 mM) at 15°C. The cross peaks a to f are assigned as follows: a – U9:NH1-A18:H2, b – G19:NH1-C8:NH4_2_, c – G19:NH1-C8:NH4_1_ d - G14:NH1-C13:NH4_2_, e - G14:NH1-C13:NH4_1_, f - G10:NH1-C17:NH4_2_, g - G10:NH1-C17:NH4_1_. **C**. Imino region of the 1D ^1^H spectra of the miR-93 recorded at various temperatures. Assignments are annotated for the imino proton resonances.
(TIF)

Figure S3 **Structural probing of 3'-end labeled (A, C) and 5'-end labeled (B, D) pre-miR-21.** **A, B, C, D**. Cleavage patterns obtained from limited hydrolysis of pre-miR-21 with RNase T1, RNase V1, and nuclease S1. Lanes: C - reaction control; L – OH ladder; T1 - limited hydrolysis with RNase T1 (0.025u/µl) in denaturing condition. **A, B**. Lines: V1 - limited hydrolysis with RNase V1 (0.0002 u/µl), S1 - limited hydrolysis with nuclease S1 (0.0095u/µl). **C, D**. Lanes: N1 - limited hydrolysis with RNase T1 (0.025 and 0.05 u/µl) in native conditions; N2 - limited hydrolysis with RNase T1 (0.025 and 0.05 u/µl) in native-magnesium conditions. Positions of RNase T1-induced digestion products are indicated in autoradiograms. **E**. Secondary structure of pre-miR21 RNA with indicated the sites of RNase T1, RNase V1 and nuclease S1 cleavage. The efficiency of pre-miR-21 cleavage is indicated by the different size of arrows.
(TIF)

Figure S4 **Analysis of RNase H1-induced cleavage of pre-miR-21 hybridized with oligodeoxyribonucleotides complement to different regions of pre-miR-21.** **A**. The sequences of oligonucleotides (O1-O8) complement to different regions of pre-miR-21. Sequence of miR-21 is marked in grey. **B**. The cleavage patterns obtained for the 5′end-labeled pre-miR-21 incubated with RNase H1 and oligodeoxynucleotides complement to pre-miR21. Lines: C - reaction control; L – OH ladder; T1- limited hydrolysis by RNase T1 (0.025u/µl) in denaturing conditions; 0 - control sample, without oligodeoxynucleotide; O1-08 – reactions with 5 µM or 10 µM antisense oligonucleotides (O1-O8) and RNase H1 (0.04u/µl). **C**. Secondary structure of pre-miR-21 and an antisense DNA (O5) (a solid line). RNase H1-induced cleavage site is indicated by arrow.
(TIF)

Figure S5 **Pb^2+^-induced hydrolysis of pre-miR-21.** **A, B**. Cleavage patterns obtained for the 3′end-labeled (A) and 5′end-labeled (B) pre-miR-21 RNA incubated with Pb^2+^. Lines: C - reaction control; L – OH ladder; T1- limited hydrolysis by RNase T1 (0.025 u/µl) in denaturing conditions. Different Pb^2+^ concentrations (0, 0.1, 0.3, 0.5, 0.7, 0.9, 1.1 mM) and positions of RNase T1-induced hydrolysis in denaturing condition are indicated, respectively above and on the right of autoradiograms. **C**.Secondary structure of pre-miR21 RNA with indicated Pb^2+^-induced cleavage sites. The efficiency of Pb^2+^-induced cleavage of pre-miR-21 RNA is indicated by the different size of arrows.
(TIF)
