# Supplementary figures and images for: Mature MiRNAs Form Secondary Structure, which Suggests Their Function beyond RISC
Source: PLoS One. 2014 Nov 25;9(11):e113848. doi: 10.1371/journal.pone.0113848 (PMC4244182; doi:10.1371/journal.pone.0113848)

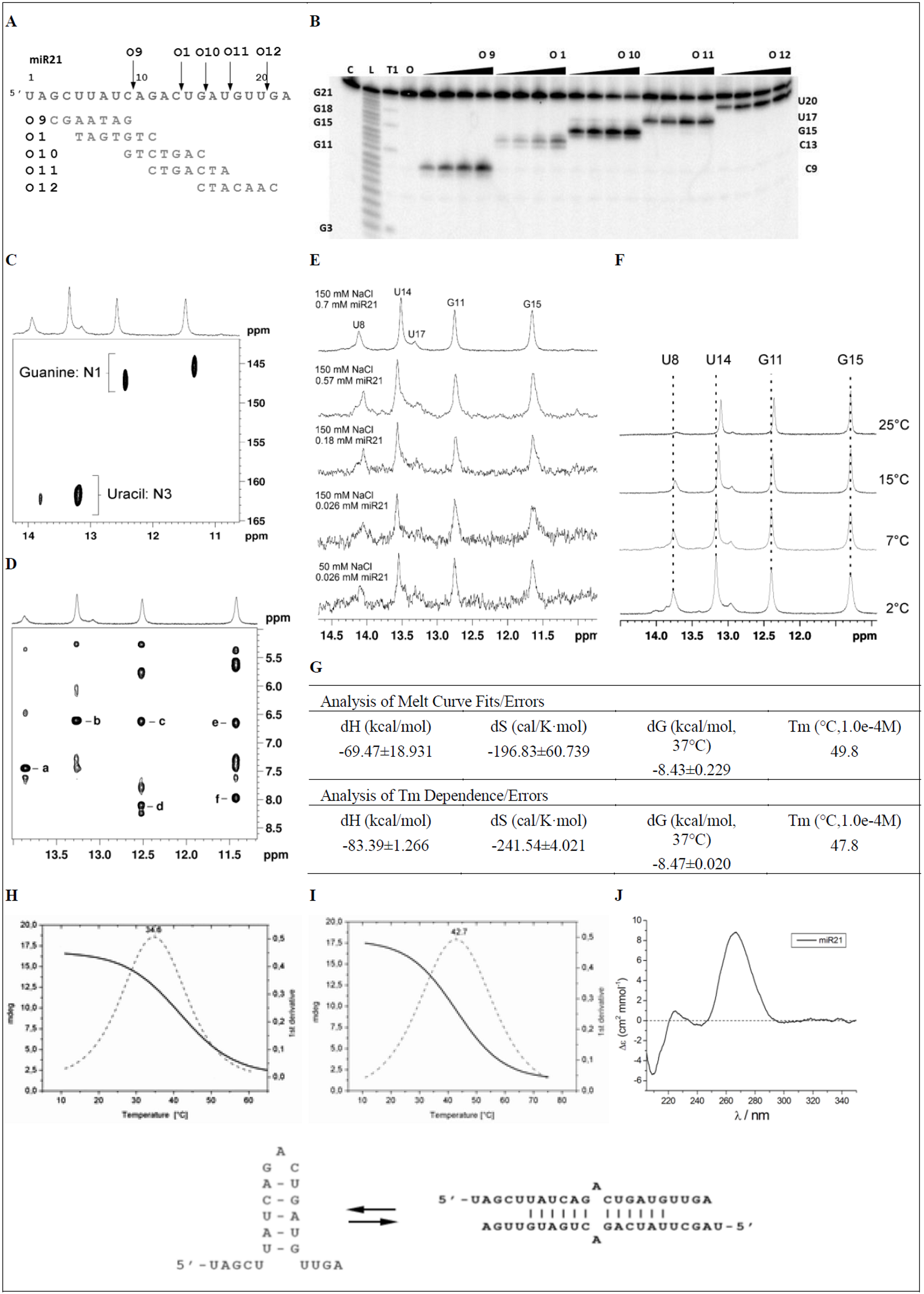

Supplement: Figure S1 — Enzymatic (A, B) probing of miR-21 structure, NMR analysis (C-F), UV (G) thermal melting and circular dichroism profile (H-J) of miR-21. A, B. Cleavage patterns obtained for limited hydrolysis of 5'-end labeled miR-21 with RNase H1 in presence of oligodeoxynucleotides complement to different regions of miR-21, and ‘hammerhead’ ribozyme. Lanes: C - reaction control; L – OH ladder; T1 - limited hydrolysis with RNase T1 (0.025 u/µl) in denaturing condition. A. The sequences of oligonucleotides (O1, O9-O12) complement to different regions of miR-21. Sequence of miR-21 is marked in grey. B. Lines O1, O9-O12 – hydrolysis with RNase H1(0.04 u/µl) in different concentrations (0, 1.25, 2.5, 5 or 10 µM) of oligonucleotides (O1, O9-O12) complement to different regions of miR-21. C. 1H-15N HSQC spectrum of the miR-21 recorded at 7°C. The assignments are indicated. D. Expanded 2D NOESY contour plots (150 ms mixing time) of miR21 (0.7 mM) at 15°C. The cross peaks a to f are assigned as follows: a – U8:NH1-A16:H2, b – U14:NH1-A10:H2, c – G11:NH1-C13:NH42 d - G11:NH1-C13:NH41, e - G15:NH1-C9:NH42, f - G15:NH1-C9:NH41. E. Imino regions of the 1D 1H spectra recorded at different RNA strand and salt concentrations. Assignments are annotated for the imino proton resonances. F. Imino region of the 1D 1H spectra of the miR-21 recorded at various temperatures. Assignments are annotated for the imino proton resonances. G. Analysis of Tm dependence over the ranges 0.8–73 µM. H, I. CD thermal melting profiles of miR-21 (4.2 µM), (H) (22.6 µM), (I) at 260 nm obtained for the temperatures between 10°C–75°C. The melting temperatures of approximately 35°C and 43°C were estimated from the first derivative. J. Circular dichroism (CD) profile of miR-21 (13.2 µM) obtained at 25°C (150 mM sodium chloride, 10 mM phosphate buffer and 0.1 mM EDTA, pH 6.6). (TIF) [file pone.0113848.s001.tif]

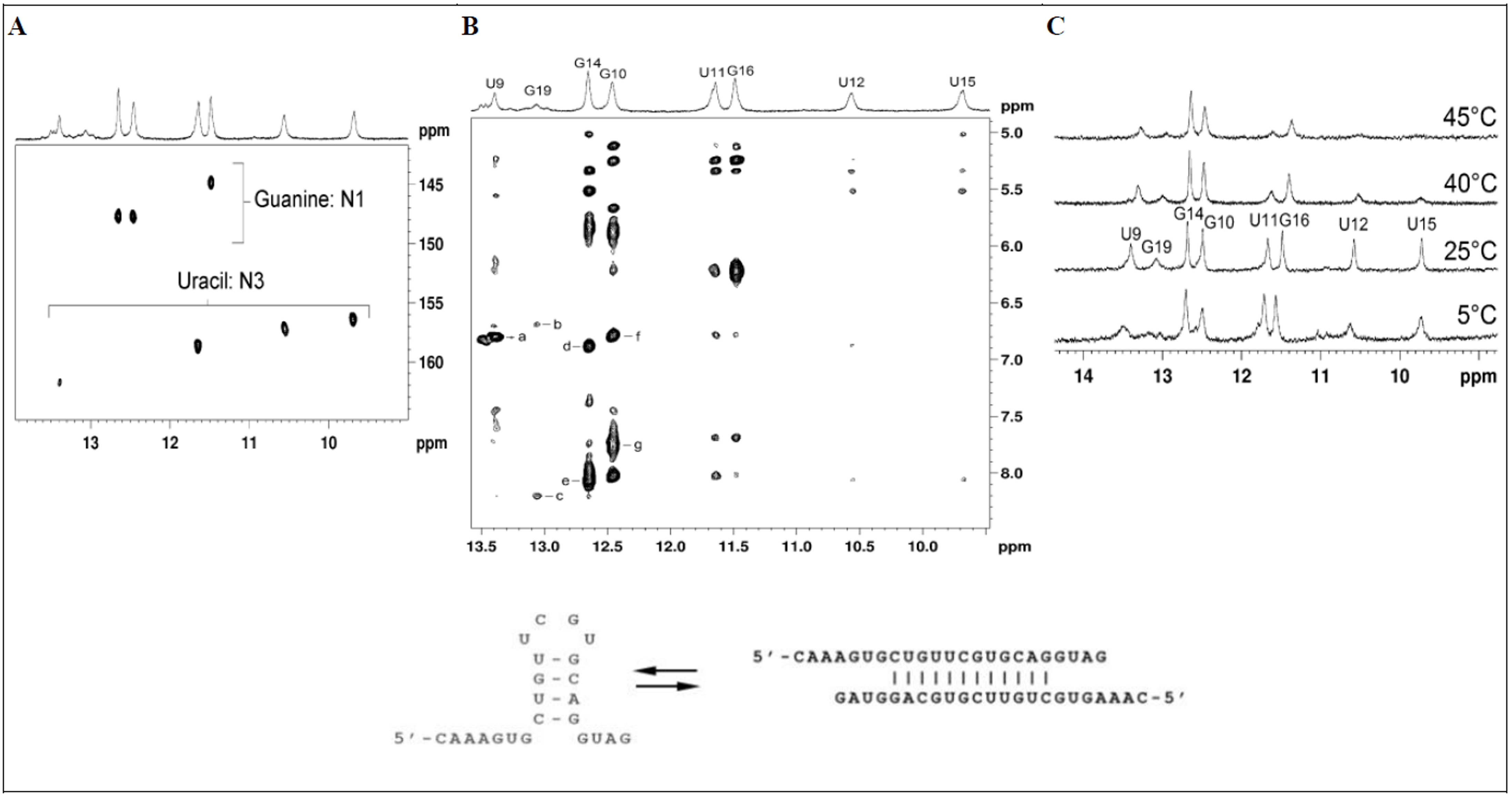

Supplement: Figure S2 — NMR analysis of miR-93. A. 1H-15N HSQC spectrum of the miR-93 recorded at 15°C. The assignments are indicated. B. Expanded 2D NOESY contour plots (150 ms mixing time) of miR-93 molecule (0.75 mM) at 15°C. The cross peaks a to f are assigned as follows: a – U9:NH1-A18:H2, b – G19:NH1-C8:NH42, c – G19:NH1-C8:NH41 d - G14:NH1-C13:NH42, e - G14:NH1-C13:NH41, f - G10:NH1-C17:NH42, g - G10:NH1-C17:NH41. C. Imino region of the 1D 1H spectra of the miR-93 recorded at various temperatures. Assignments are annotated for the imino proton resonances. (TIF) [file pone.0113848.s002.tif]

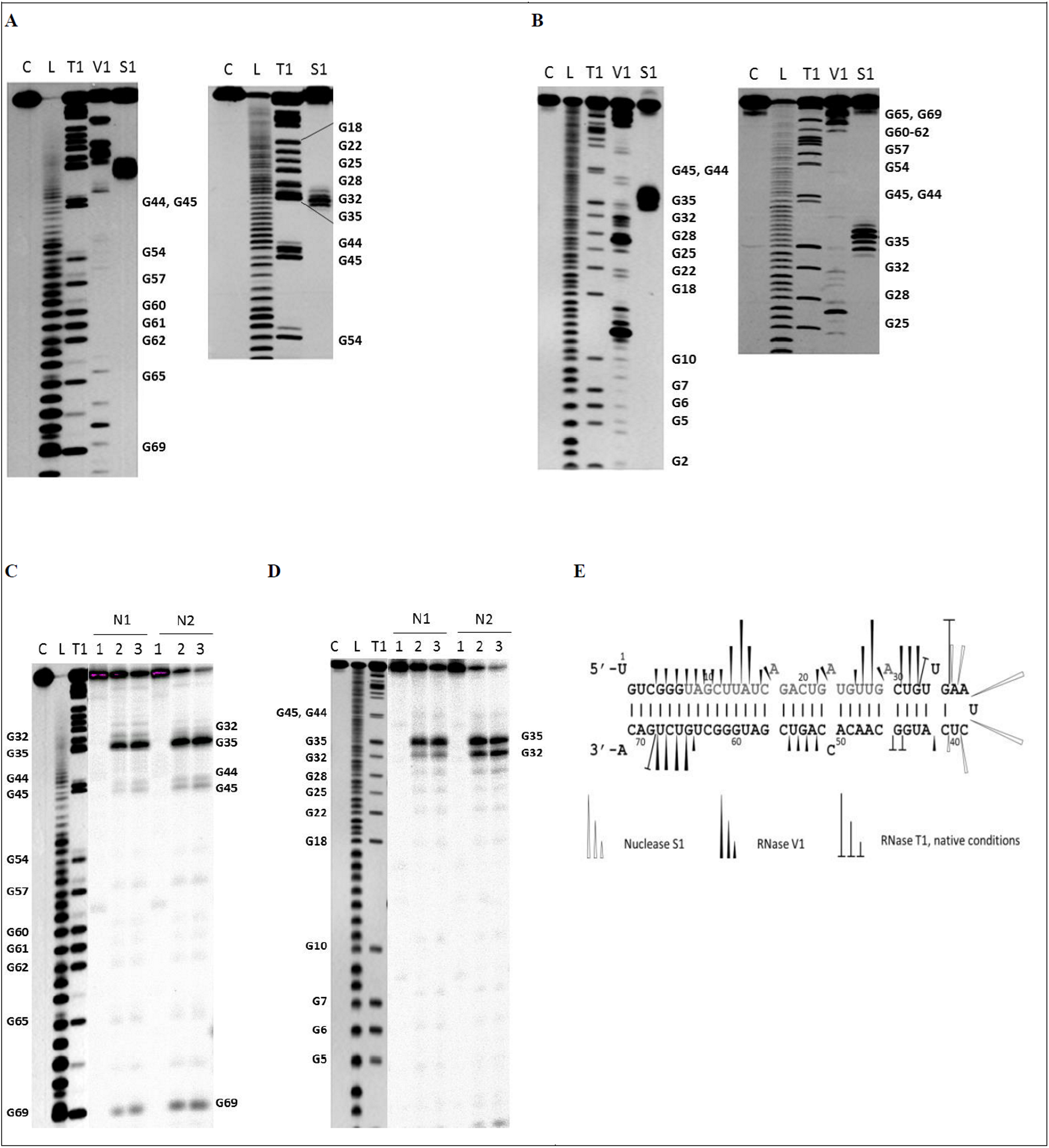

Supplement: Figure S3 — Structural probing of 3'-end labeled (A, C) and 5'-end labeled (B, D) pre-miR-21. A, B, C, D. Cleavage patterns obtained from limited hydrolysis of pre-miR-21 with RNase T1, RNase V1, and nuclease S1. Lanes: C - reaction control; L – OH ladder; T1 - limited hydrolysis with RNase T1 (0.025u/µl) in denaturing condition. A, B. Lines: V1 - limited hydrolysis with RNase V1 (0.0002 u/µl), S1 - limited hydrolysis with nuclease S1 (0.0095u/µl). C, D. Lanes: N1 - limited hydrolysis with RNase T1 (0.025 and 0.05 u/µl) in native conditions; N2 - limited hydrolysis with RNase T1 (0.025 and 0.05 u/µl) in native-magnesium conditions. Positions of RNase T1-induced digestion products are indicated in autoradiograms. E. Secondary structure of pre-miR21 RNA with indicated the sites of RNase T1, RNase V1 and nuclease S1 cleavage. The efficiency of pre-miR-21 cleavage is indicated by the different size of arrows. (TIF) [file pone.0113848.s003.tif]

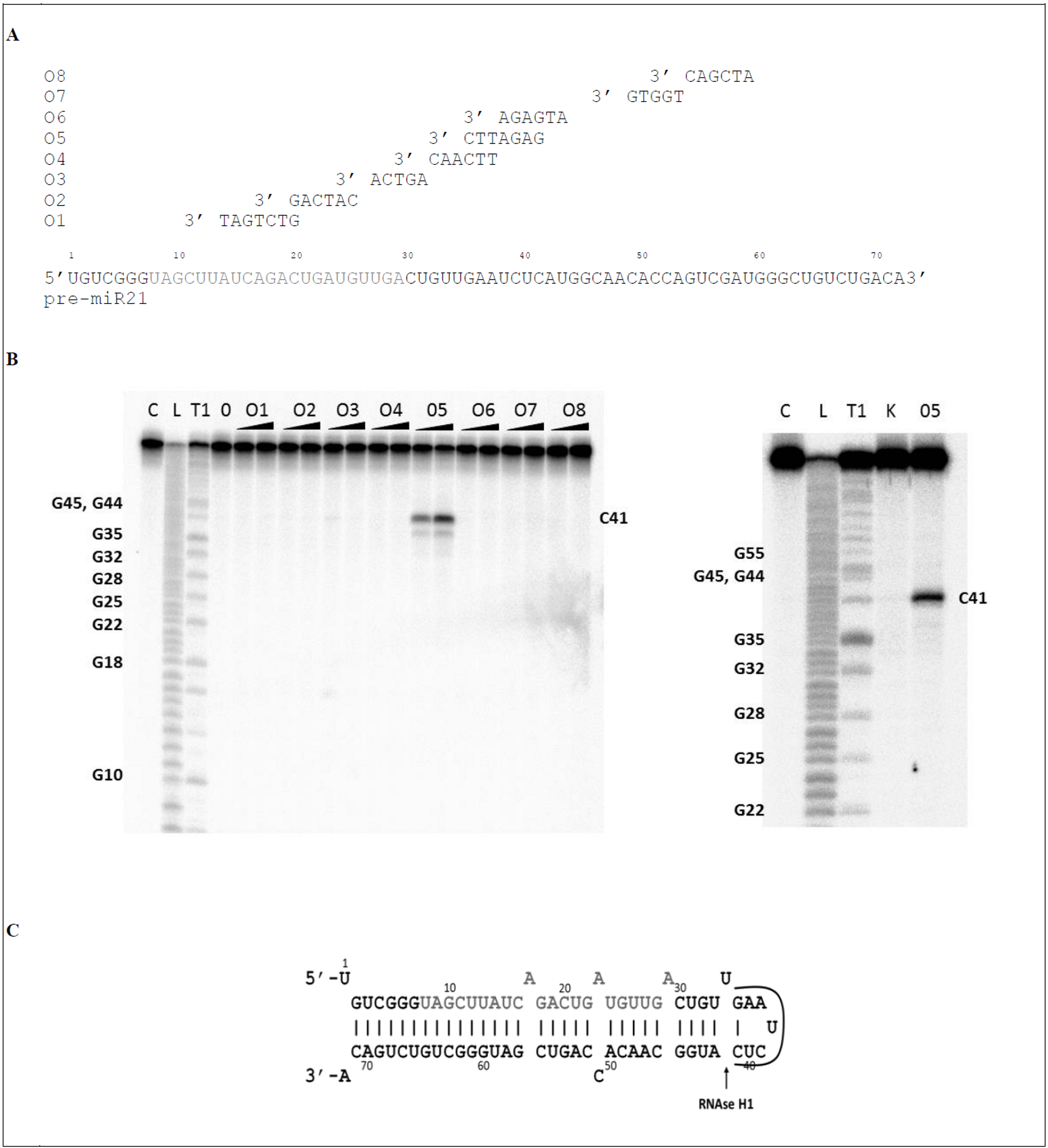

Supplement: Figure S4 — Analysis of RNase H1-induced cleavage of pre-miR-21 hybridized with oligodeoxyribonucleotides complement to different regions of pre-miR-21. A. The sequences of oligonucleotides (O1-O8) complement to different regions of pre-miR-21. Sequence of miR-21 is marked in grey. B. The cleavage patterns obtained for the 5′end-labeled pre-miR-21 incubated with RNase H1 and oligodeoxynucleotides complement to pre-miR21. Lines: C - reaction control; L – OH ladder; T1- limited hydrolysis by RNase T1 (0.025u/µl) in denaturing conditions; 0 - control sample, without oligodeoxynucleotide; O1-08 – reactions with 5 µM or 10 µM antisense oligonucleotides (O1-O8) and RNase H1 (0.04u/µl). C. Secondary structure of pre-miR-21 and an antisense DNA (O5) (a solid line). RNase H1-induced cleavage site is indicated by arrow. (TIF) [file pone.0113848.s004.tif]

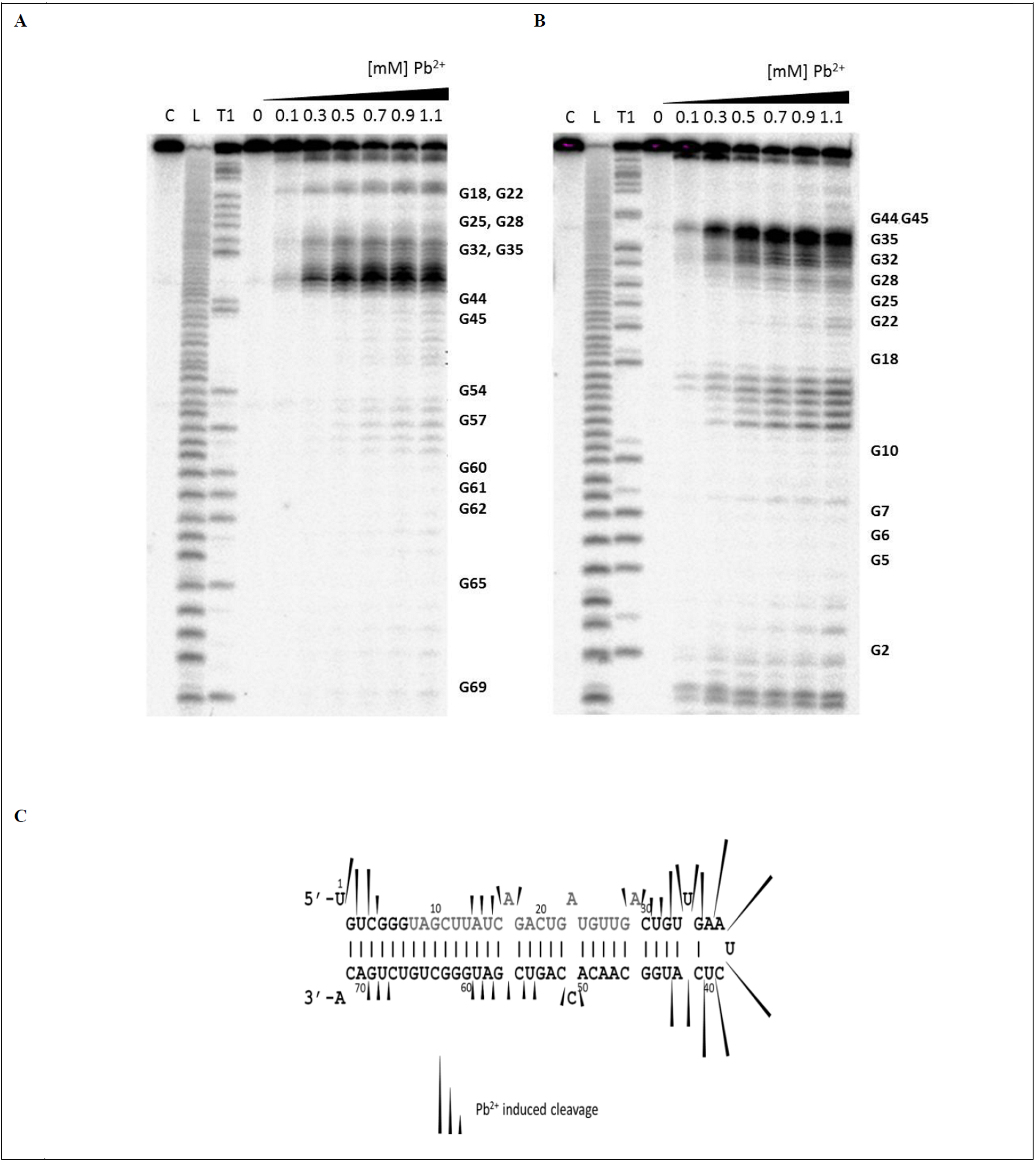

Supplement: Figure S5 — Pb2+-induced hydrolysis of pre-miR-21. A, B. Cleavage patterns obtained for the 3′end-labeled (A) and 5′end-labeled (B) pre-miR-21 RNA incubated with Pb2+. Lines: C - reaction control; L – OH ladder; T1- limited hydrolysis by RNase T1 (0.025 u/µl) in denaturing conditions. Different Pb2+ concentrations (0, 0.1, 0.3, 0.5, 0.7, 0.9, 1.1 mM) and positions of RNase T1-induced hydrolysis in denaturing condition are indicated, respectively above and on the right of autoradiograms. C.Secondary structure of pre-miR21 RNA with indicated Pb2+-induced cleavage sites. The efficiency of Pb2+-induced cleavage of pre-miR-21 RNA is indicated by the different size of arrows. (TIF) [file pone.0113848.s005.tif]
